# Involvement of YAP, TAZ and HSP90 in Contact Guidance and Intercellular Junction Formation in Corneal Epithelial Cells

**DOI:** 10.1371/journal.pone.0109811

**Published:** 2014-10-07

**Authors:** Vijay Krishna Raghunathan, Britta Dreier, Joshua T. Morgan, Binh C. Tuyen, Brad W. Rose, Christopher M. Reilly, Paul Russell, Christopher J. Murphy

**Affiliations:** 1 Department of Surgical and Radiological Sciences, School of Veterinary Medicine, University of California Davis, Davis, CA, United States of America; 2 Department of Pathology, Microbiology & Immunology, School of Veterinary Medicine, University of California Davis, Davis, CA, United States of America; 3 Department of Ophthalmology & Vision Science, School of Medicine, University of California Davis, Davis, CA, United States of America; Cedars-Sinai Medical Center; UCLA School of Medicine, United States of America

## Abstract

The extracellular environment possesses a rich milieu of biophysical and biochemical signaling cues that are simultaneously integrated by cells and influence cellular phenotype. Yes-associated protein (YAP) and transcriptional co-activator with PDZ-binding motif (*WWTR1*; TAZ), two important signaling molecules of the Hippo pathway, have been recently implicated as nuclear relays of cytoskeletal changes mediated by substratum rigidity and topography. These proteins intersect with other important intracellular signaling pathways (e.g. Wnt and TGFβ). In the cornea, epithelial cells adhere to the stroma through a 3-dimensional topography-rich basement membrane, with features in the nano-submicron size-scale that are capable of profoundly modulating a wide range of fundamental cell behaviors. The influences of substratum-topography, YAP/TAZ knockdown, and HSP90 inhibition on cell morphology, YAP/TAZ localization, and the expression of TGFβ2 and CTGF, were investigated. The results demonstrate (a) that knockdown of TAZ enhances contact guidance in a YAP dependent manner, (b) that CTGF is predominantly regulated by YAP and not TAZ, and (c) that TGFβ2 is regulated by both YAP and TAZ in these cells. Additionally, inhibition of HSP90 resulted in nuclear localization and subsequent transcriptional-activation of YAP, formation of cell-cell junctions and co-localization of E-cadherin and β-catenin at adherens junctions. Results presented in this study reflect the complexities underlying the molecular relationships between the cytoskeleton, growth factors, heat shock proteins, and co-activators of transcription that impact mechanotransduction. The data reveal the importance of YAP/TAZ on the cell behaviors, and gene and protein expression.

## Introduction

The corneal epithelium plays a central role in corneal homeostasis and vision by maintaining a protective tight junctional barrier and by transmitting and refracting light rays to the retina. The cornea is continuously subjected to physical, chemical, and biological stimuli from the external environment which can result in disruption of epithelial integrity and loss of barrier function. Healing of a corneal epithelial defect involves the coordination of a number of complex processes including cell migration, cell proliferation and differentiation, as well as matrix (basement membrane) deposition and remodeling. Dysregulation of these processes can result in chronic or recurrent epithelial defects and loss of transparency due to excessive fibrosis and haze formation [1]–[3]. In corneal epithelial wounds in which the basement membrane (BM) has been removed, re-establishment of the BM is a critical process, promoting epithelial adhesion, migration, proliferation, differentiation, and reformation of adherens junctions [4].

*In vivo*, extracellular matrix (ECM) proteins such as laminin, collagen and fibronectin are among the protein constituents that comprise the rich 3D topographic landscape for the corneal epithelial cells. While the biochemical makeup of the ECM is well known to regulate numerous key cellular functions [5]–[7], research from our laboratory and others has consistently demonstrated that biophysical cues are as potent as soluble signaling molecules in determining fundamental corneal cell behaviors such as cellular adhesion, migration, proliferation and response to growth factors [8]–[12]. Although numerous studies have evaluated the influence of topographical features on changes in actin and cytoskeleton dynamics, a knowledge gap exists regarding critical early steps in the translation of external biophysical cues into biochemical events.

Recent reports have identified Yorkie homologues Yes-associated protein (YAP) and transcriptional coactivator with PDZ-binding motif (TAZ; encoded by *WWTR1*) as nuclear relays of mechanical signals exerted by substrate rigidity and geometry [13]–[15]. Their role in mechanotransduction entails the modulation of cytoskeleton (actin stress fibers) and activation of Rho [13]–[15]. Phosphorylation appears to be the primary method of regulating YAP and TAZ (YAP/TAZ). Unphosphorylated YAP/TAZ are targeted to the nucleus where they function as co-factors of transcription by their interaction with TEAD, Runx or the SMAD proteins [16]–[19]. When phosphorylated, YAP/TAZ are sequestered in the cytoplasm by 14-3-3σ and targeted for degradation. In addition to their transcriptional co-activity, they interact with proteins of the TGFβ (transforming growth factor β) and Wnt pathways including SMADs and Disheveled [20]–[22]. TGFβ and Wnt signaling have been shown to play crucial roles in regulating corneal epithelial differentiation [23], [24], wound healing [25] and transition from the epithelial to the mesenchymal phenotype (EMT) [26], [27]. Recent work in vision science has revealed the importance of YAP/TAZ in the ocular tissues of the trabecular meshwork, lens and retina [28]–[36], but there is a paucity of information regarding their role in corneal epithelial biology.

It is well documented that anisotropic topographical features induce corneal epithelial cell elongation and alignment mediated by alterations to the cytoskeleton [8], [11], [37]–[39]; however, the specific influence of topography on the expression and intracellular spatial localization of YAP/TAZ and subsequent transcriptional activation is unknown. In this study, the influence of substratum topographic features in modulation of YAP/TAZ and associated genes and the specific role of YAP/TAZ in influencing cell alignment and formation of cell-cell junctions (a key attribute of a healthy epithelium) was investigated. The impact of altered spatial localization of YAP/TAZ was further explored by inhibition of HSP90. 17-Allylamino-17-demethoxygelanamycin (17-AAG), a drug that directly inhibits heat shock protein 90 (HSP90) with subsequent depletion of the LATS1/2 kinases, which phosphorylate YAP/TAZ, was used for these studies [40]. Suppression of phosphorylation of YAP/TAZ results in nuclear localization of YAP/TAZ and thus promotes its transcriptional function. The influence of such spatial modulation of YAP/TAZ and subsequent effect on their transcriptional targets and ECM regulatory genes [connective tissue growth factor (CTGF) and transforming growth factor β2 (TGFβ2)] was investigated in the presence and absence of topographic cues. Additionally, we investigated alterations to proteins modulated by cytoskeletal remodeling (ERK1/2) and those essential to the formation of cell-cell junctions (E-cadherin & β-catenin).

## Materials and Methods

### 2.1 Fabrication of polymeric topographically patterned substrates

Patterned silicon chips containing ridge and groove features were fabricated using X-ray lithography as previously described [11]. The silicon masters were fabricated either containing an array of 6 (2×2 mm) areas with pitches of 400, 800, 1200, 1600, 2000 and 4000 nm separated by planar control areas; termed “6-packs”, or larger surfaces (6.5 cm^2^) possessing a single pitch of 400, 1400 or 4000 nm as well as chemically identical planar surfaces; termed “monotypic”. The larger monotypic surfaces are required for harvesting sufficient material for gene and protein studies while the 6 packs are used for determining the phenotypic consequences of differing size scale features. The dimensions of the various topographic features were such that ridges and grooves were of equal width with groove depth of 300 nm. A composite stamp of the silicon chip master was made by curing a “hard” layer of poly (dimethylsiloxane) (PDMS) to retain the topographic features and then a pliable PDMS layer for easy removal and handling of the stamp. The pattern could then be replicated into a thin layer of NOA81 (Norland Products, Cranbury, NJ) optical adhesive [41] deposited onto 35 mm (for 6-packs) or 60 mm (for monotypics) tissue culture plates using a spin coater and cured in a XL-1500 UV cross-linker under 365 nm light for 100 minutes. NOA81, a proprietary mercapto-ester compound of Norland Products, is supplied as a single component liquid adhesive that readily cures as a rigid polymer with exposure to UV light. Research from our laboratory has previously demonstrated NOA81 as a suitable material for cell culture [38], [42], [43].

### 2.2 Preparation of substrates for cell culture

All NOA81 substrates were sterilized by exposure to 280 nm UV light for 30 min in a laminar flow hood. Prior to cell seeding, a molecular coating of FNC (Athena Enzyme Systems, Baltimore, MD) was applied to all surfaces.

### 2.3 Culture of immortalized human corneal epithelial cells

All work with human tissue was done in accordance with the tenets of Helsinki. Donor human corneoscleral rims with no history of corneal diseases that were unsuitable for transplant were used to establish primary epithelial cultures as previously described [9], [38]. Human cadaver corneas were graciously donated by the Lions Eye Bank of Wisconsin, Madison or the Missouri Lions Eye Bank (Columbia, MO). These tissue samples obtained were not human subjects research and as such approval from committee or kin were not necessary. Briefly, sclera and limbal regions of the cornea were trimmed and the tissue was immersed in dispase (1.2 U/ml, Boehringer, Mannheim, Germany) at 37°C for 4 h. Corneal epithelial cells were removed by gently rubbing the anterior surface with a sterile pipette tip. Cell suspensions were pooled, centrifuged and re-suspended in EpiLife medium (Life Technologies, Carlsbad, CA) supplemented with EpiLife defined growth supplement (EDGS; Life Technologies) and 1% penicillin/streptomycin (Life Technologies) and used between passages 2 and 3. Experiments were repeated with cells isolated from three different donors.

Immortalized human corneal epithelial cells (hTCEpi; [44]), kindly provided by Dr James V Jester (UC Irvine), were maintained in EpiLife medium as above and were used between passages 40–60.

### 2.4 Inhibition of HSP90 and subsequent nuclear translocation of YAP/TAZ by treatment with 17-N-Allylamino-17-demethoxygeldanamycin (17-AAG)

Nuclear translocation of YAP/TAZ was achieved by treating hTCEpi cells with 17-N-Allylamino-17-demethoxygeldanamycin (17-AAG). Briefly, cells were seeded and allowed to attach overnight. The dose of 17-AAG required for nuclear translocation of YAP without eliciting toxicity or gross alterations to cellular morphologically was empirically determined by MTT (Sigma-Aldrich, St. Louis, MO) viability assay [45]. Cells were treated with 45 nM 17-AAG for 24 h, and cells were harvested for RNA or protein isolation, or fixed appropriately for immunocytochemistry.

### 2.5 siRNA transfection of hTCEpi cells

YAP and TAZ were knocked down in hTCEpi cells using siRNAs targeted to YAP1 (Hs_YAP1_5; Qiagen, Valencia, CA) and TAZ (WWTR1; Hs_WWTR1_1; Qiagen). CTGF was knocked down using siRNA targeted to CTGF (FlexiTube GeneSolution GS1490 for CTGF; Qiagen). Transfection efficiencies were determined against AllStars Negative control siRNA (Qiagen). All transfections were performed in hTCEpi cells (60–80% confluent) using HiPerfect transfection reagent (Qiagen) following manufacturer’s instructions with final siRNA concentration of 50 nM. 48 h after transfection, RNA was isolated using RNeasy kit (Qiagen). In all experiments, knockdown of mRNA expression levels to below 20% was achieved as validated by quantitative real-time PCR analyses using aptamers specific to YAP1 (Hs00371735_m1), TAZ (WWTR1; Hs00210007_m1), and CTGF (Hs00170014_m1).

### 2.6 RNA isolation and quantitative Real-time PCR analyses

To determine mRNA expression levels in cells cultured on planar or topographically patterned surfaces, cells were harvested for RNA isolation 15 h after plating on FNC coated surfaces (unless specified otherwise). RNA was isolated using the RNeasy kit (Qiagen) following manufacturer’s instructions. Equal amounts of RNA (75 ng) were used for all qPCR reactions. Expression levels of YAP1, TAZ, connective tissue growth factor (CTGF) and transforming growth factor 2 (TGFβ2) were determined using the SensiFAST Probe Hi-ROX One-Step Kit (Bioline USA Inc, Taunton, MA) and aptamers specific to YAP1, TAZ, CTGF or TGFβ2 (Hs00234244_m1), all from Life Technologies. The reverse transcription reaction was performed in a StepOne qPCR machine (Applied Biosystems/Life Technologies) with the following parameters: 30 minutes at 50°C followed by 10 minutes at 95°C; forty cycles of 60°C for 1 minute followed by 95°C for 15 seconds. Relative expression levels of the genes of interest were normalized to the expression of GAPDH (Hs99999905_m1; Life Technologies). The experiment was performed in triplicate and repeated three times.

### 2.7 Protein isolation and Western blotting

Cell monolayers cultured on NOA81 surfaces were washed once in PBS and lysed and scraped into RIPA buffer (Thermo Scientific, Waltham, MA) supplemented with protease and phosphatase inhibitors (Fisher Scientific, Hampton, NH) on ice. The cells were homogenized and centrifuged at 1000 g for 1 min to remove any cell debris. Protein was quantified using a modified Lowry assay (DC assay, Bio-Rad, Hercules, CA) with bovine serum albumin as the standard. Protein homogenate was then denatured in Laemmli buffer (Sigma-Aldrich, St. Louis, MO) by boiling for 10 min. Approximately 10 µg protein per well were loaded for each sample. Protein was separated on NuPAGE 10% Bis-Tris precast gels and transferred onto nitrocellulose membranes (both Life Technologies). Membranes were incubated overnight, 4°C, in blocking buffer (0.3% gelatin in PBS-Tween). Immunoblotting was done against anti-human YAP (H-9; Santa Cruz Biotechnologies, Santa Cruz, CA), pYAP (S-127) (Cell Signaling Technologies, Danvers, MA), TAZ (H70; Santa Cruz Biotechnologies), HSP90 (Cell Signaling Technologies) and beta-actin (Abcam, Cambridge, MA) overnight at 4°C in blocking buffer. This was followed by incubation with secondary antibodies conjugated with horseradish peroxidase (HRP; Kirkegaard & Perry Laboratories, Gaithersburg, MD, USA) for 1 h at 37°C. Protein bands were detected by chemiluminescence using ECL plus Western Blotting detection kit (GE Life Sciences, Pittsburgh, PA). Blots were imaged using an ImageQuant 350 imaging system (GE Life Sciences). Densitometry analysis of the protein bands was performed using NIH ImageJ [46].

### 2.8 Immunocytochemistry

hTCEpi cells were fixed in 4% formaldehyde in phosphate buffered saline (pH 7.4; 20 min), permeabilized in 0.1% Triton X-100 for 5 min, and blocked in PBS buffer containing 0.3% gelatin for 1 h, 37°C. Cells were then incubated with phalloidin-AlexaFluor 594 (Life Technologies) or antibodies specific to YAP-H125 (Santa Cruz BioTechnologies), TAZ-H70 (Santa Cruz BioTechnologies), E-cadherin (Novus Biologicals, Littleton, CO) or β-catenin (BD Signal Transduction Laboratories) for 2 h, 37°C in blocking buffer containing 0.1% sodium azide and 0.3% gelatin. Cells were washed three times in PBS and subsequently incubated with secondary antibodies conjugated with AlexaFluor 488 or 594 (Life Technologies) for 1 h at 37°C in blocking buffer. Nuclei were stained using DAPI (Life Technologies) and cells were imaged using an Axiovert 200 M inverted epifluorescent microscope (Carl Zeiss, Germany).

### 2.9 Immunohistochemistry

The corneoscleral rim from donors with no history of ocular disease was used. Tissue samples from at least three donors were used to verify the presence of YAP/TAZ expression in corneal epithelium. Data from only one donor (51 year old) is shown since it was representative of the results from all donors. A strip of tissue from edge to edge across the central cornea, approximately 2 mm wide, was dissected and fixed overnight in 10% neutral buffered formalin, paraffin embedded and sectioned. Sections were deparaffinized in xylene, subjected to citrate antigen retrieval, peroxidase blocked, and incubated overnight at 4°C with mouse anti-human YAP-H9 (Santa Cruz Biotechnologies, Santa Cruz, CA) and TAZ (Abnova, Walnut, CA) antibodies. Sections were then treated with horse anti-mouse biotinylated secondary antibody, followed by streptavidin-horseradish peroxidase, and developed with Vector Red chromogen prior to counterstaining with hematoxylin and coverslipping.

### 2.10 Junctional analysis

Cells treated with 17-AAG or vehicle control were fixed and stained for β-catenin and E-cadherin as described above. 5–7 fields of ∼50% confluent cells were imaged and presence of junctional staining was ranked on a scale of 1–4 by a masked observer. The scores were averaged between the fields. Data is presented as the mean ± SEM of 3 independent replicates.

### 2.11 Orientation/alignment analyses

Alignment of cells to underlying anisotropic ridges and grooves were determined as previously described [8], [47]. Control or siRNA transfected cells were plated at a density of 15,000 cells/cm^2^ in 35 mm dishes containing two 6-pack polyurethane substrates with topographic features. After overnight incubation, cells were fixed and stained for filamentous actin using phalloidin as described above. The orientation of the cytoskeleton of individual cells in relation to the underlying pitch was determined from fluorescent images using Carl Zeiss AxioVision software. Cells included for analysis had to be fully contained within the border of the image, not in physical contact with other cells, and not undergoing mitosis. The orientation of the cells was based on the angle between the major axis of the object and the underlying feature. Cells were considered aligned parallel with the ridges and grooves when this angle was between 0° and 10° and perpendicular when the angle was between 80° and 90°. Cell elongation is defined as the ratio between the length and breadth of each cell. Cells were considered elongated if this factor was >1.3. A total of 100–250 cells for a given pitch size in 4 replicates were analyzed.

### 2.12 Statistical analysis

All statistical analyses were performed using SigmaPlot software (SYSTAT software Inc, San Jose, CA). Multiple comparisons between respective control (planar or control siRNA transfected cells) and experimental groups were performed using one-way ANOVA or t-test. Kruskal-Wallis multiple comparison test was performed in place of ANOVA if normal distribution test failed. In all graphs statistically significant differences between control and 17-AAG treated/siRNA transfected cells for a given pitch or planar area are marked using the following symbols (or as indicated in specific figure legends): ###p<0.001, ##p<0.01, #p<0.05. Statistically relevant differences between cells grown on planar surfaces and cells exposed to a given topographic pitch are marked as followed: ***p<0.001, **p<0.01, *p<0.05.

## Results

### 3.1 YAP and TAZ are expressed in the human cornea

**Figure 1:**
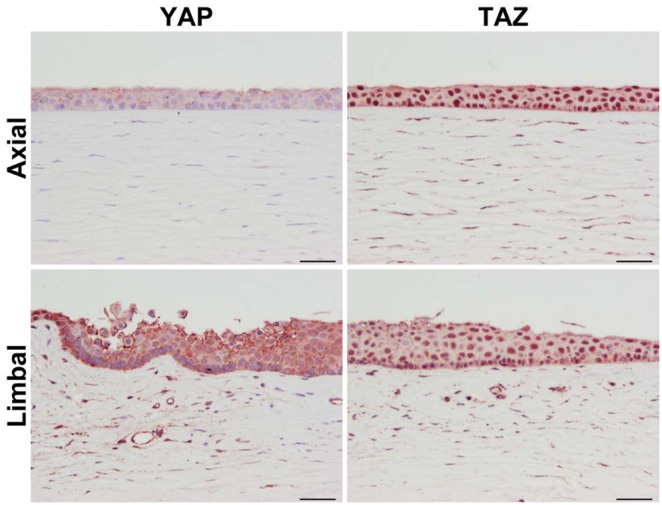
Representative image demonstrating differential expression of YAP and TAZ in human corneal epithelium. **(Left panels)** Expression of YAP in both the axial and limbal corneal epithelial sections was cytoplasmic for this donor. Stromal cells stained weakly for YAP. **(Right panels)** Expression of TAZ on the other hand was predominantly nuclear both in the axial and limbal regions of the corneal epithelium. Scale bar represents 50 µm.

To determine whether YAP and TAZ were expressed *in vivo*, immunohistochemical staining for YAP/TAZ on human corneas was performed. We observed weak cytoplasmic YAP staining in corneal epithelial cells located axially with very limited staining of stromal cells (Figure 1A). Conversely, TAZ was strongly expressed in both the epithelium and stroma with mixed nuclear and cytoplasmic localization (Figure 1B). In the limbal region, suspected to be the stem cell niche for corneal cells [48], [49], YAP staining dramatically increased with a mixture of cytoplasmic and junctional staining (Figure 1C), while TAZ staining decreased slightly, and was predominantly nuclear (Figure 1D). These data suggest that YAP and TAZ may play distinctive roles in maintaining corneal homeostasis.

### 3.2 Substratum topographic cues differentially regulate CTGF and TGFβ2 via YAP/TAZ

**Figure 2:**
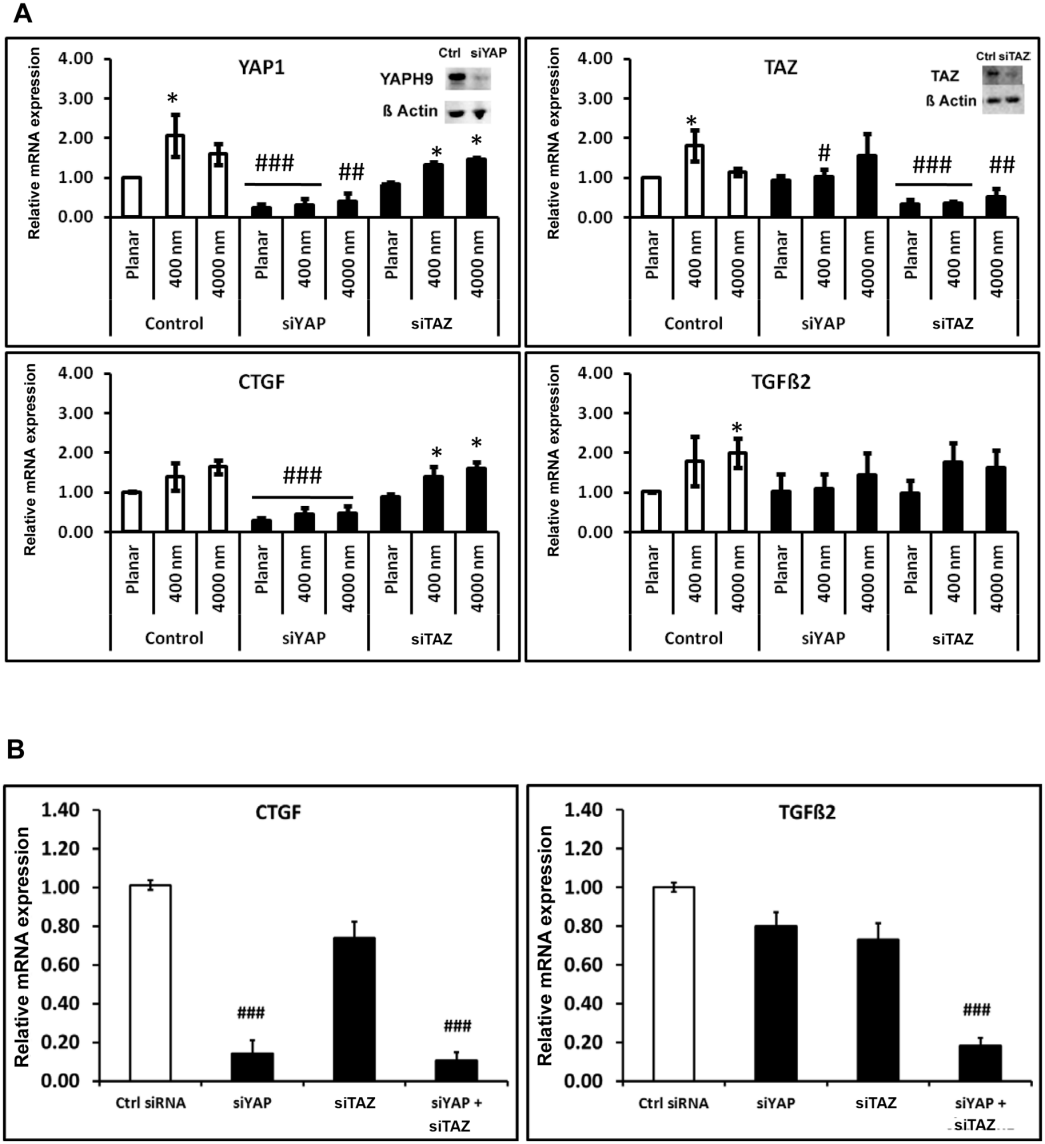
Interaction of YAP & TAZ and their modulation of TGFβ2 and CTGF in immortalized corneal epithelial cells (hTCEpi). (**A**) Knockdown of YAP did not alter mRNA expression of TAZ and knockdown of TAZ did not alter the mRNA expression of YAP indicating they do not moderate each other’s expression. No specific trends were observed for TGFβ2 mRNA expression after YAP or TAZ were individually knocked down. CTGF mRNA expression was inhibited after singular knockdown of YAP. Experiments were performed three times and a minimum of three reactions were run for each sample. Figure insets are Western blots demonstrating knockdown of YAP and TAZ on the protein level. (**B**) Simultaneous knockdown of YAP and TAZ inhibits TGFβ2 and CTGF mRNA expression in immortalized corneal epithelial cells. Results are mean ± standard deviation, n = 3 reactions. Statistical comparisons were made by ANOVA followed by Dunnett’s multiple comparison test. *p<0.05 compared with planar control, ##p<0.01, ###p<0.001 compared with control non-targeted siRNA.

To determine the influence of substratum topographic cues on the expression and localization of YAP/TAZ in hTERT-immortalized human corneal epithelial (hTCEpi) cells, qPCR and immunocytochemical analyses were performed. hTCEpi cells were plated on chemically identical topographically patterned substrates of parallel ridges and grooves with pitches (ridge width + groove width) of 400 or 4000 nm as well as planar surfaces. A significant increase in mRNA expression of both YAP and TAZ was observed on surfaces with biomimetic size-scale topography (400 nm *vs* planar; Figure 2A) compared with planar surfaces. Additionally, two of their transcriptional targets (CTGF and TGFβ2) trended towards being upregulated on biomimetic pitches (400 nm *vs* planar; Figure 2A) though the difference did not reach statistical significance. On the largest scale features investigated (4000 nm pitch) TGFβ2 mRNA was significantly upregulated (*vs* planar; Figure 2A). No significant differences were observed in the spatial localization of YAP or TAZ in these cells on any pitch (data not shown) suggesting that substratum topography does not influence their spatial localization in these cells. Additionally, no significant differences in protein expression for YAP, pYAP or TAZ were observed between the different surfaces (Figure S1). However it is possible that the changes in protein were subtle and below detection level using Western blots when compared with changes in mRNA level detected by qPCR. Similar results were obtained when experiments were repeated using primary corneal epithelial cells (data not shown).

In order to better elucidate the roles of YAP and TAZ in mediating the increased expression of CTGF and TGFβ2 on biomimetic topography, YAP and TAZ were individually knocked-down using siRNAs in hTCEpi cells. The specificity and efficiency of knockdown was determined by qPCR (Figure 2A) and Western blotting (Insets, Figure 2A). Knockdown efficiencies of at least 80% were obtained up to 72 h after siRNA transfection (Figure 2A). Additionally, nonspecific knockdown of TAZ was not observed with siRNA for YAP, and vice versa. In TAZ knockdown cells on 400 nm and 4000 nm topography, YAP expression was upregulated in comparison with cells on planar surfaces. After knockdown of YAP but not TAZ, CTGF expression was significantly decreased (<30% of control). The expression profile of CTGF mirrored the expression of YAP on both topographically patterned and planar substrates. Also, double knockdown of YAP and TAZ resulted in sustained inhibition of CTGF expression in these cells (Figure 2B) comparable to the inhibited expression observed after YAP knockdown. mRNA expression of TGFβ2 was minorly altered with knockdown of either YAP or TAZ. To test whether YAP or TAZ were individually sufficient to maintain normal TGFβ2 expression, we performed simultaneous knockdown of both YAP and TAZ and observed a significant decrease in the TGFβ2 expression (Figure 2B).

### 3.3 Knockdown of YAP/TAZ and contact guidance of corneal epithelial cells

**Figure 3:**
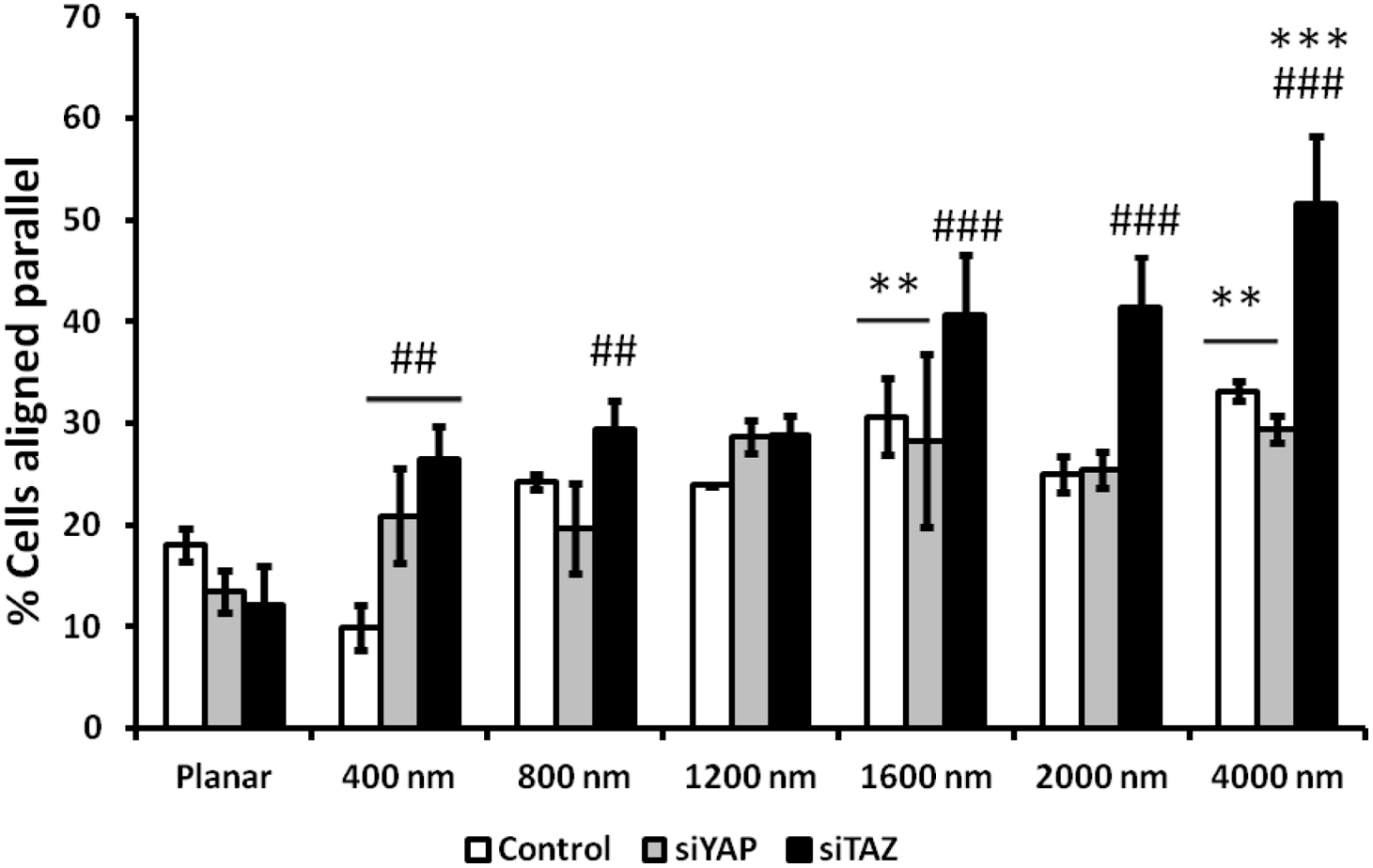
Downregulation of TAZ but not YAP mRNA expression increased cell alignment to topography. No statistical significance was observed in cell alignment between planar and 400 nm pitch surfaces for control siRNA group. Cell alignment was significantly upregulated after TAZ knockdown with the underlying ridges and grooves greater than 1600 nm with greatest number of cells aligned on 4000 nm pitch. Orientations of at least 200 cells per experiment per pitch were analyzed. Results were expressed as mean ± standard error of the mean of n = 3 individual experiments. Statistical comparisons were performed using Kruskal-Wallis pairwise multiple comparison, **p<0.01, ***p<0.001 compared with planar, ##p<0.01, ###p<0.001 compared with control non-targeted siRNA.

To determine the effect of YAP/TAZ downregulation on the response of hTCEpi cells to substratum topographic cues, cell alignment with respect to the underlying parallel ridges and grooves was determined (Figure 3). As expected on planar surfaces, control, YAP siRNA and TAZ siRNA transfected cells were oriented in a random manner. Alignment of cells on pitches >800 nm were comparable (i.e. 25–35% of all cells aligned with the long axis of the ridges and grooves) for control and YAP siRNA transfected cells. Interestingly, on pitch sizes >1600 nm, a significantly greater number of TAZ siRNA transfected cells (40–60%; p<0.001) aligned with the long axis of the underlying ridges and grooves in comparison with control or YAP siRNA transfected cells. No statistical differences were observed in cell alignment between planar and 400 nm pitch patterned topographies for control siRNA treated cells.

### 3.4 Inhibition of HSP90 results in nuclear translocation of YAP/TAZ and increased expression of transcriptional targets CTGF and TGFβ2

**Figure 4:**
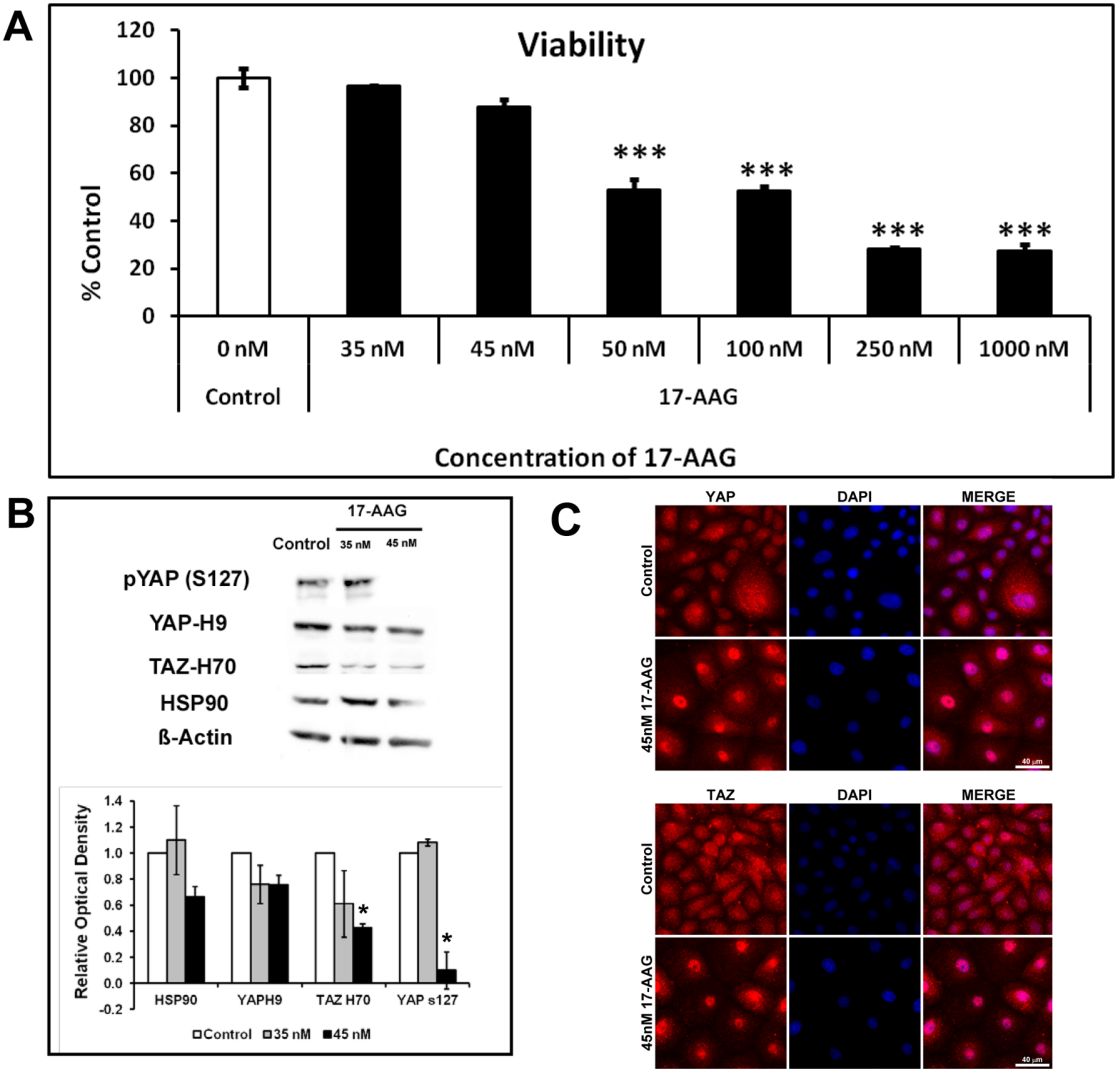
Treatment with 17-N-Allylamino-17demethoxygeldanamycin (17-AAG, an inhibitor of HSP90) resulted in nuclear localization of YAP/TAZ in immortalized corneal epithelial cells. (**A**) Toxicity of 17-AAG to hTCEpi cells was observed at doses ≥50 nM. (**B**) Treatment with 45 nM of 17-AAG inhibited the protein expression of HSP90, TAZ and phospho-YAP (Ser172) in these cells without influencing the expression of total YAP. Blots are representative of data obtained from three individual experiments and graph demonstrating relative optical density are mean ± standard deviation from 3 experiments. (**C**) Reduction in cytoplasmic distribution accompanied by increased nuclear localization of YAP/TAZ was more apparent in cells treated with 45 nM 17-AAG (red channel for YAP/TAZ and blue channel for nucleus). Statistical comparisons were performed using one-way Kruskal-Wallis multiple comparison, ***p<0.001 compared with control (0 nM).

**Figure 5:**
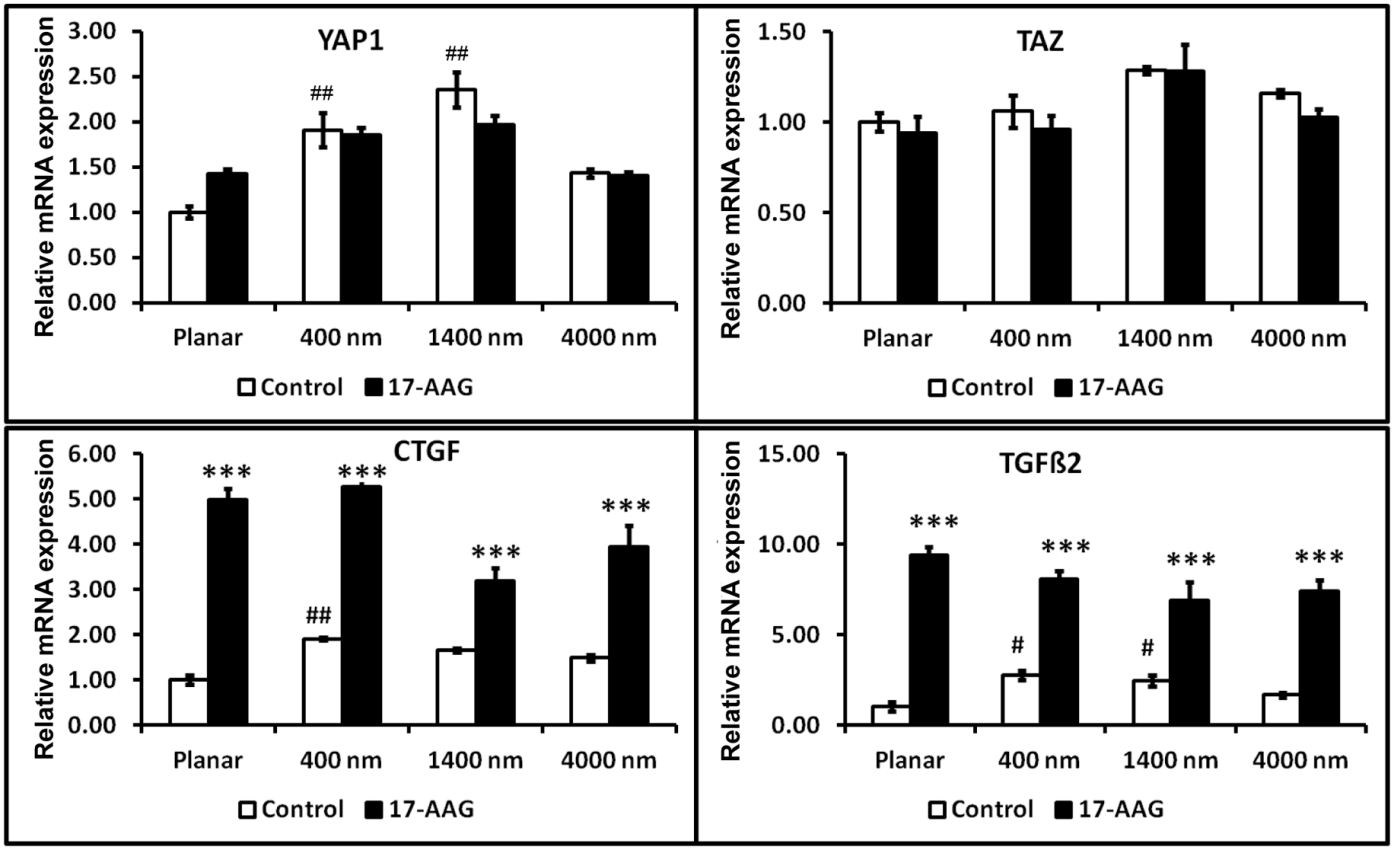
Treatment with 17-N-Allylamino-17demethoxygeldanamycin (17-AAG) resulted in upregulation of CTGF and TGFβ2 mRNA, but not of YAP or TAZ in immortalized corneal epithelial cells. The effect of HSP90 mediated regulation of CTGF and TGFβ2 were independent of topographic cues. The experiment was performed three times and at least 3 reactions were run for each sample. Results were expressed as mean ± standard deviation, n = 3 reactions. Statistical comparisons were performed using one-way ANOVA followed by Dunnett’s multiple comparison test; ***p<0.001 compared with DMSO control cells and ##p<0.01 compared with cells on planar surfaces.

In order to understand the influence of substratum topography on ECM gene expression regulated by YAP/TAZ in hTCEpi cells, cytoplasmic localization of YAP/TAZ was inhibited using a small molecule inhibitor of HSP90, 17-AAG. Loss of cell viability was greater than 50% when cells were treated for 24 h with 17-AAG concentrations of ≥50 nM (Figure 4A). Therefore for subsequent experiments we used the maximum permissible dose that was non-toxic to cells but resulted in changes in YAP localization i.e. 45 nM. Significant inhibition of TAZ (p<0.05) and phospho YAP (pYAP-S127; p<0.05) was observed in hTCEpi cells treated with 45 nM 17-AAG for 24 h, while YAP expression was not significantly affected (p>0.1; Figure 4B). Although not statistically significant (p = 0.058), expression of HSP90 trended to be inhibited in cells treated with 45 nM 17-AAG. Consistent with loss of phosphorylation, YAP and TAZ exhibited increased nuclear localization (Figure 4C). This was accompanied by a significant increase in mRNA expression of YAP/TAZ transcriptional targets, CTGF and TGFβ2, but this was independent of the pitch size (Figure 5).

**Figure 6:**
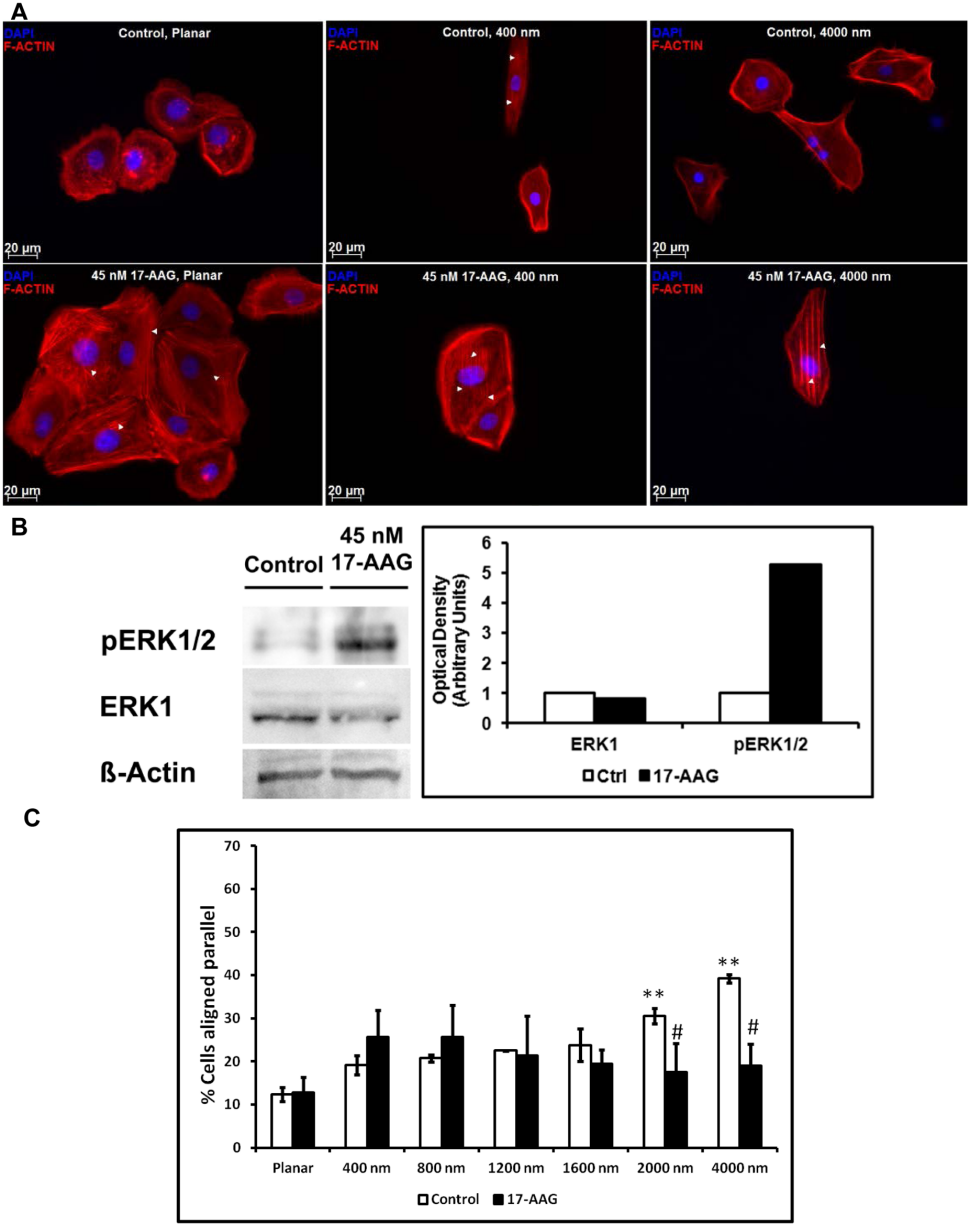
Treatment with 17-AAG resulted in increased formation of stress fiber in immortalized corneal epithelial cells. (**A**) Treatment of hTCEpi cells with 45 nM 17-AAG for 24 h resulted in elevated formation of stress fibers on both planar and topographically patterned surfaces (indicated by white arrowheads). The occurrence of stress fibers was most pronounced in cells cultured on 4000 nm pitch surfaces. (**B**) Treatment with 45 nM 17-AAG increased pERK1/2. However, it did not significantly alter expression of ERK1. (**C**) The percentage of cells aligned along the microscale (2000 and 4000 nm) pitched ridges and grooves was significantly decreased in comparison with control cells. Statistical comparisons were performed using Kruskal-Wallis pairwise multiple comparison, #p<0.01 compared with untreated control, **p<0.01 compared with planar. Although the sample images suggest changes in cell morphology, there were no documented statistically significant changes in cell area.

**Figure 7:**
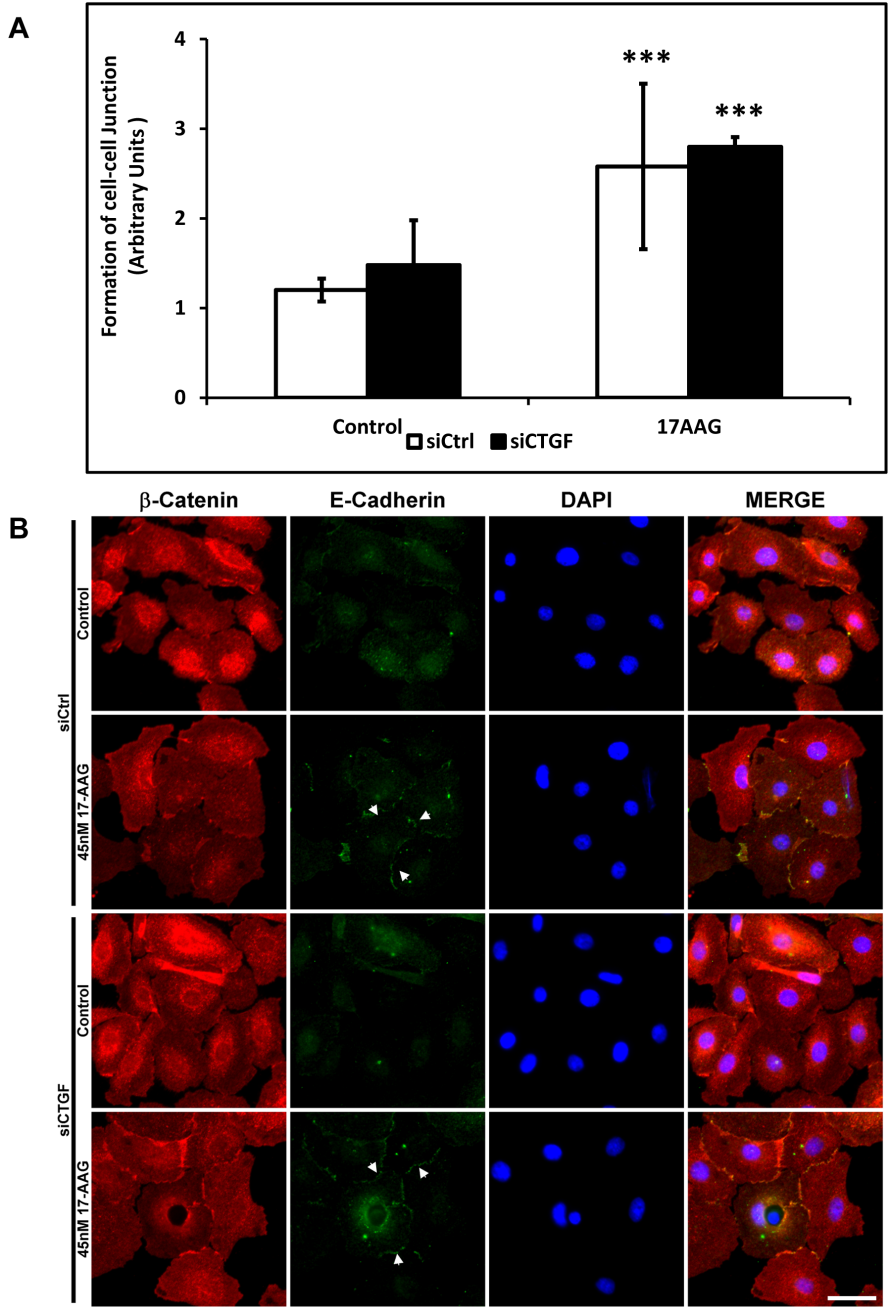
Treatment with 17-AAG resulted in increased incidence of formation of cell-cell junctions accompanied by co-localization of E-cadherin/β-catenin in cell-cell junctions independent of CTGF expression in immortalized corneal epithelial cells. (**A**) Formation of cell junctions in control (DMSO vehicle) versus 17-AAG treated cells was determined by ranking on a scale of 1–4 (1: Low incidence of cell junction, 4: High incidence of cell junction) by a masked observer. Results were expressed as mean ± standard error of the mean (***p<0.001 compared with control, t-test). (**B**) Co-localization of β-catenin (Red) and E-cadherin (Green) was observed at the cell-cell junctions (white arrows) both in siCtrl and siCTGF cells after 17-AAG treatment. Scale bar is 20 µm.

Treatment with 17-AAG also induced the formation of stress fibers in cells cultured on planar and all topographically patterned (400–4000 nm) surfaces (Figure 6A). This change was most apparent in cells cultured on patterned surfaces with the fibers aligning parallel to the ridges and grooves and was accompanied by a strong up-regulation of pERK1/2 with no change in total ERK1 levels (Figure 6B). Despite increased alignment of the stress fibers, treatment resulted in significantly fewer cells aligning parallel with the ridges and grooves on pitches greater than 2000 nm. Interestingly, after 17-AAG treatment, the cells formed significantly greater number of cell-cell adhesions (Figure 7A) with E-cadherin and ß-catenin co-localizing to the cell-cell junctions (Figure 7B). To determine if 17-AAG facilitated formation of cell-cell junctions was dependent on CTGF [50], we performed CTGF knockdown and measured co-localization of β-catenin to cell junction. Formation of cell-cell junctions after 17-AAG treatment was unaltered in DMSO control or 17-AAG treated siCTGF cells (Figure 7A).

### 3.5 Simultaneous influence of TAZ knockdown, YAP/TAZ double knockdown, and 17-AAG on contact guidance of corneal epithelial cells

**Figure 8:**
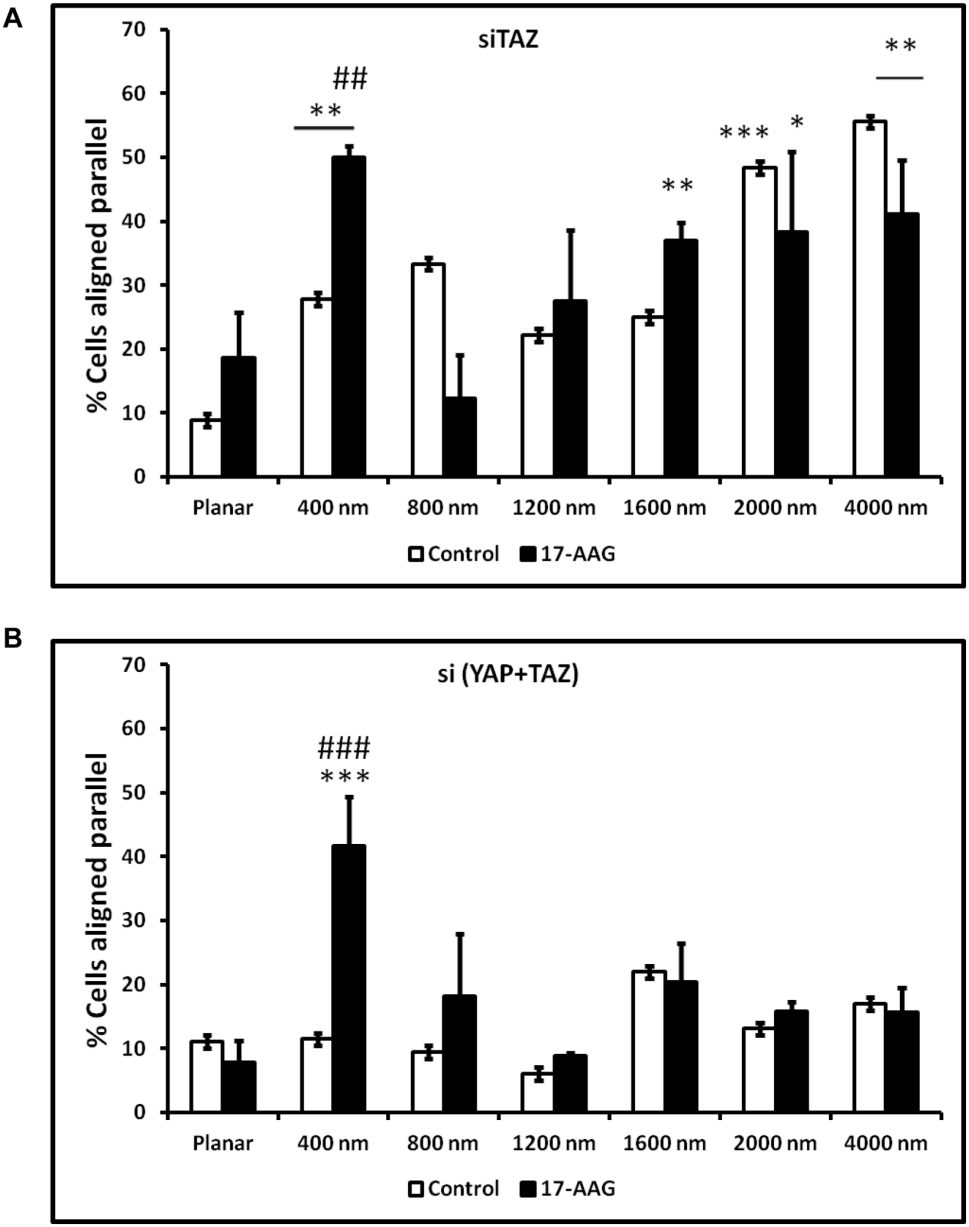
Simultaneous effects of 17-AAG treatment and TAZ knockdown or YAP/TAZ double knockdown. (**A**) Treatment of TAZ knockdown cells with 17-AAG facilitated significant increases in cell alignment only when cultured on 400 nm surfaces and remained unaltered when compared with control cells on other pitches. (**B**) Double knockdown of YAP/TAZ eliminates contact guidance in corneal epithelial cells. However, treatment of double knockdown cells with 17-AAG resulted in a significant increase in cell alignment only on 400 nm pitch. Statistical comparisons were performed using Kruskal-Wallis pairwise multiple comparison, *p<0.05, **p<0.01, ***p<0.001 compared with planar and ##p<0.01, ###p<0.001 compared with control cells.

Since TAZ knockdown but not YAP knockdown increased cell alignment and treatment with 17AAG significantly inhibited cell alignment, we decided to investigate further the interaction between YAP/TAZ and HSP90 inhibition in controlling contact guidance of hTCEpi cells. We combined silencing TAZ and double knockdown of YAP/TAZ with 17-AAG treatment for this experiment. Double knockdown combined with 17-AAG treatment significantly decreased cell viability (60% of control) in hTCEpi cells (Figure S2). After TAZ silencing, 17-AAG treatment did not significantly reduce contact guidance (Figure 8A), in contrast to TAZ-expressing cells (Figure 6C). Indeed, we observed a significant increase in alignment of siTAZ cells on the 400 nm pitch with 17-AAG treatment (Figure 8A). We then knocked down both YAP and TAZ and found no significant change in alignment on any surface without 17-AAG (Figure 8B; control). Similar to siTAZ, 17-AAG treatment again caused a dramatic increase of alignment on the 400 nm topography in the double knock down cells (Figure 8B).

## Discussion

The current study demonstrates that YAP and TAZ are expressed in human corneal epithelial cells and that a differential expression is observed at the limbus. Substratum topographic features in the biomimetic size scale were found to influence the gene expression of mechanotransducers, YAP/TAZ, and their transcriptional targets, CTGF & TGFβ2 in corneal epithelial cells. However, topographic cues presented without modulation of the biochemical signaling environment, did not alter spatial localization or protein expression of YAP/TAZ in corneal epithelial cells.

While YAP/TAZ have recently been reported to act as nuclear relays of substratum biophysical cues, their role in contact guidance is poorly understood. Contact guidance has previously been investigated in numerous cell types including but not limited to mesenchymal stem cells [51], [52], fibroblasts [53], trabecular meshwork cells [54], vascular endothelial cells [55], [56], osteoblasts [57], and neuronal cells [58], [59]. In most mesenchymal cell types, alignment along the parallel set of ridges and grooves is greater than 80%. Interestingly, contact guidance in corneal epithelial cells is significantly lower and the molecular mechanisms that underlie this partial response in the cell population have not been determined. Data from our lab have previously shown that approximately 30% of primary or immortalized corneal epithelial cells align along the ridges and grooves [11], [37], [38], [47], [60], [61]. The alignment response of epithelial cells can be altered differentially by controlling the scale of topographic features [8], [47], [60], depth of the grooves [62], spacing of the ridges and grooves [11], [39], [63], the presence or absence of surface ECM proteins [43], spacing of arginine-glycine-aspartic acid (RGD) moieties [8], [64], [65] and by knockdown of focal adhesion kinase [37].

Data presented in this study demonstrate the relationship between YAP, TAZ and the response to topographic cues by corneal epithelial cells is complex. TAZ knockdown but not YAP knockdown increased the percentage of corneal epithelial cells aligned parallel with anisotropically ordered topographic cues (Figure 3). Simultaneous knockdown of both YAP and TAZ induced a complete abrogation of contact guidance (Figure 8B; control). Together, these data suggest that expression of either YAP or TAZ is sufficient to maintain contact guidance, but that TAZ can limit YAP-mediated contact guidance. A corollary experiment to modulate the extent of contact guidance after YAP/TAZ over expression would need to be performed and is a subject for future studies.

CTGF and TGFß2, two transcriptional targets of YAP/TAZ, have critical functions in the eye and other organs. A rich body of evidence documents the importance of CTGF during development and tissue remodeling in various cell types [66]–[70] and association of CTGF with fibronectin enhances attachment and migration of corneal epithelial cells [71], [72]. CTGF has been reported to be regulated by both YAP [19], [40] and TAZ [18]. In our experiments, knockdown of YAP but not TAZ inhibited the expression of CTGF demonstrating that YAP regulates CTGF in corneal epithelial cells. When YAP and TAZ were both simultaneously knocked down expression CTGF was inhibited to a similar extent observed with YAP knockdown alone, suggesting that YAP is essential for the maintenance of CTGF in corneal epithelial cells.

TGFβ signaling also plays multiple roles in the anterior segment of the eye: TGFβ1 plays a predominant role in the differentiation of keratocytes to myofibroblasts [12], [73], TGFβ2 is immunosuppressive in normal human and rabbit aqueous humor [74], [75] and is a key cytokine that can influence corneal wound healing [76]. In this study, no alteration to TGFß2 was observed with separate knockdown of either YAP or TAZ. However, its expression was dramatically inhibited when both YAP and TAZ were simultaneously knocked down. In their unphosphorylated state and associated nuclear localization, both YAP and TAZ interact with TEAD resulting in transcription of TGFß2 [77], albeit with differences in their mode of interaction [78]. Our data suggest that both YAP and TAZ can act independently and each is sufficient to maintain expression of TGFß2. We speculate that YAP and TAZ may have distinct differential transcriptional activity, as demonstrated by sustained expression of TGFβ2 but not of CTGF, when either YAP or TAZ is knocked down.

Intracellular localization of YAP/TAZ largely determines their functional consequence. Inhibition of HSP90 was previously demonstrated to deplete LATS1/2 [40], thus decreasing phosphorylation of YAP/TAZ and subsequent cytoplasmic localization [22]. Results presented in this study (Figure 4C) clearly demonstrate nuclear translocation of YAP/TAZ after treatment with 17-AAG, thereby facilitating their transcriptional activity as evidenced by significant elevation of CTGF and TGFβ2 mRNA expression. Treatment of corneal epithelial cells with 17-AAG also increased the formation of stress fibers, consistent with previous reports [79], but did not promote cell alignment on any scale of topographic features investigated (Figure 5). The increased formation of stress fibers was present in cells cultured on both planar and patterned surfaces with the direction of the stress fibers aligning parallel to the underlying ridges and grooves on patterned surfaces. Formation of stress fibers has previously been reported to inhibit the Hippo pathway upstream of or at LATS thus promoting nuclear YAP localization [14]. Our findings of nuclear localization of YAP (induced by 17-AAG) and a concomitant increase in actin stress fiber formation support an interaction between spatial localization of YAP and stress fiber formation.

The relationship between contact guidance, YAP/TAZ and 17-AAG is complex. As stated previously, cells expressing YAP/TAZ exhibit a statistically significant increase in cell alignment on the largest scale topographic features investigated (2000 and 4000 nm pitch) compared to other pitches examined. Additionally, this alignment response was accentuated by TAZ (but not YAP) knockdown. 17-AAG treatment, promoted nuclear localization of both YAP and TAZ (Figure 4) and inhibited the increased alignment to topographic cues only for control cells grown on 2000 and 4000 nm pitches (i.e. this effect was not observed for TAZ knockdown cells; Figures 6C and 8A). The effect of 17-AAG treatment on YAP knockdown cells was not investigated in our studies because we had not observed any effect of YAP knockdown on cell alignment.

The relationship between YAP and contact guidance is complicated and requires interaction with TAZ. Simultaneous knockdown of YAP and TAZ in control cells eliminated all contact guidance, regardless of pitch. Surprisingly, YAP/TAZ double knockdown cells treated with 17-AAG lost all contact guidance except on the smallest scale pitch (400 nm) patterned surfaces. Indeed, on 400 nm pitch surfaces cell alignment was observed to increase with simultaneous YAP & TAZ knockdown in conjunction with 17-AAG treatment. The 400 nm pitch patterned surfaces possess topographic features in the biomimetic range, being of a similar size scale to those described for the corneal epithelial BM [80]–[82]. These findings suggest that HSP90 differentially governs cell alignment to biomimetic size scale cues that is independent of YAP and TAZ but that YAP and TAZ can in some way counteract as this effect requires double knockdown. This may potentially be mediated by the increased Rho/ROCK activation after HSP90 inhibition [79], which has previously been implicated in contact guidance [83]. Whether this phenomenon would be reversed when YAP or TAZ is overexpressed remains to be investigated. It must be noted that 17-AAG is not specific to modulation of YAP/TAZ only and may thus have secondary effects within the cell mediated through HSP90. Unfortunately, a small molecule compound that *specifically* targets the phosphorylation state and thus intracellular spatial localization of YAP and/or TAZ has not been identified to date.

Our data support an association between YAP/TAZ and the maintenance of cell junctions. The association of cadherins and catenin in the adherens junction to form and maintain a polarized epithelial junctional barrier is well established [84]–[87]. Binding of β-catenin and E-cadherin at the cell junction is indicative of the antagonism of the Wnt pathway restricting nuclear translocation of β-catenin [88]. In our experiments, 17-AAG treatment resulted in the increased formation of cell-cell junctions in hTCEpi cells as shown in Figure 7. These findings are consistent with previous reports that found HSP90 depletion (using 17-AAG specifically) resulted in potent inhibition of epithelial-mesenchymal transition [89], [90].

**Figure 9:**
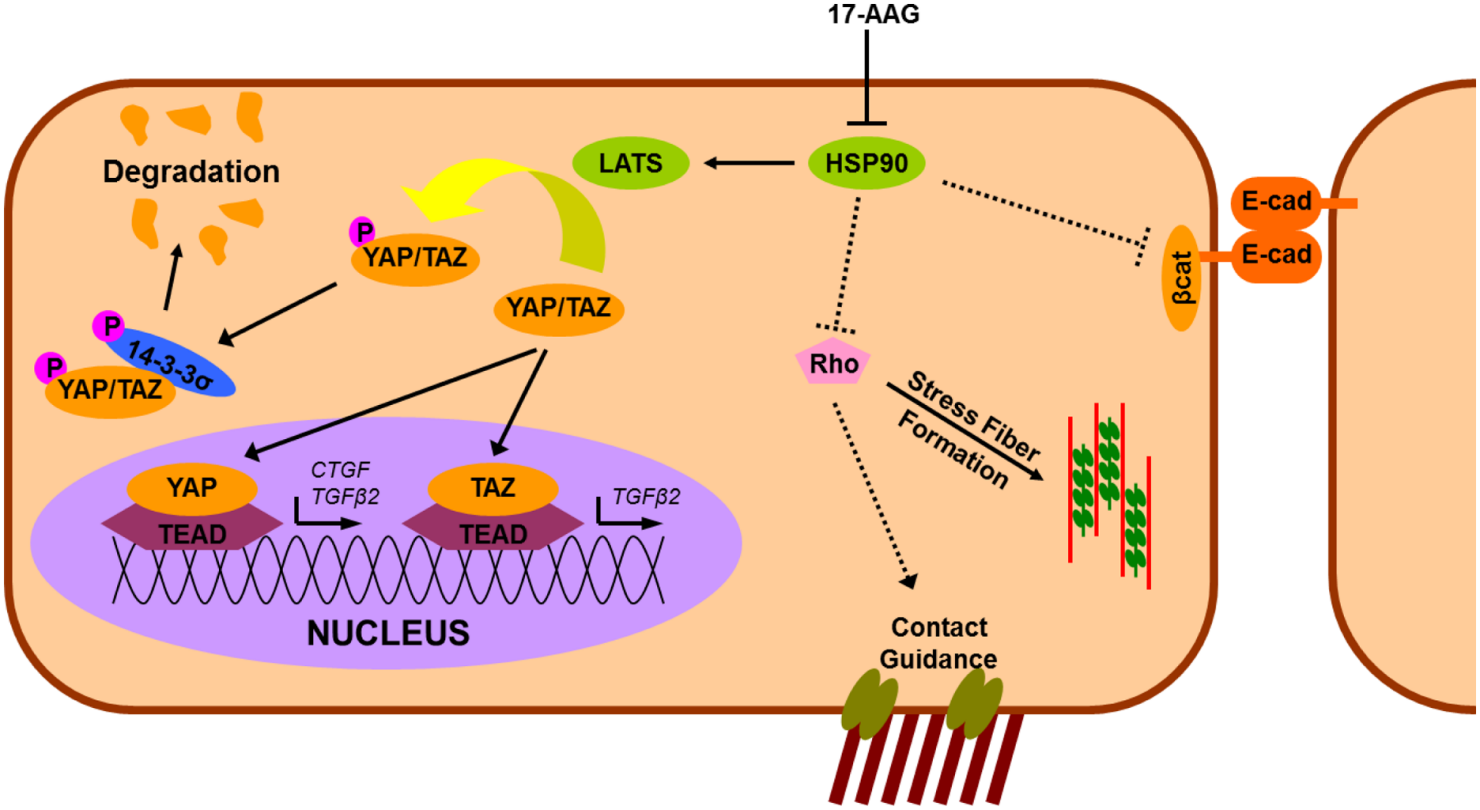
Summary of findings illustrating the differential influence of YAP/TAZ on contact guidance of corneal epithelial cells. CTGF is regulated by YAP but not TAZ in these cells, while TGFβ2 is regulated by both YAP/TAZ possibly mediated by their interactions with TEAD. Treatment of cells with 17-AAG resulted in nuclear localization of YAP/TAZ associated with E-cadherin and β-catenin localizing at cell junctions, and formation of stress fibers but losses of cell alignment on anisotropic topographical features. Contact guidance is partially regulated by both YAP and TAZ. HSP90 appears to play a role in restricting response to biomimetic size scale features that is abrogated by YAP and TAZ. Solid arrows indicate a positive regulation, while solid perpendicular lines indicate a negative regulation. Dotted lines (arrows/perpendicular lines) indicate possible mechanisms of action.

Our findings also suggest that YAP affects the dynamics of cell junction formation independent of modulation of CTGF expression as evidenced by the formation of cell-cell junctions accompanied with localization of E-cadherin and β-catenin. In contrast, in renal epithelial cells, increased expression of CTGF has been associated with increased E-cadherin expression thus facilitating the formation of adherens junctions, independent of TGFβ stimulation [50]. In the cornea, E-cadherin in epithelial cells is important as its appearance has been reported to coincide with the assembly of the BM [4]. Whether HSP90 prevents formation of cell-cell junction or if nuclear translocation of YAP or TAZ is essential in mediating formation of cell junctions is unclear at this point. Further studies that document the cross-talk between HSP90 and the Hippo pathway are therefore essential. In aggregate (Figure 9), these results suggest that corneal epithelial cells, when treated with 17-AAG, (a) increase formation of cell-cell junctions (possibly accelerating restoration of barrier function), and (b) are positioned to initiate extracellular matrix deposition evidenced by the elevated levels of CTGF/TGFβ2. Extrapolating our *in vitro* findings to *in vivo* applications, we hypothesize that compounds that regulate YAP/TAZ expression and/or their localization may represent a novel class of therapeutics for promoting epithelial wound healing by enhancing formation of cell junctions and restoring barrier function.

In aggregate, our data document complex inter-relationships between YAP, TAZ, corneal epithelial contact guidance and gene/protein expression (Figure 9). In the proposed model, we identify that treatment of corneal epithelial cells with 17-AAG promotes the nuclear translocation of YAP/TAZ possibly mediated via inhibition of HSP90 and LATS1/2 while simultaneously promoting cell-cell junction formation mediated via β-catenin/E-cadherin interactions. It is known that contact guidance is, at least partially, mediated by Rho family of proteins [83], a critical regulator of cytoskeletal dynamics. We speculate that the formation of stress fibers with inhibition of contact guidance after 17-AAG treatment may partially be mediated by the Rho-MAPK axis of proteins, although the specific role of HSP90 or YAP/TAZ mediated changes to the Rho family of proteins in contact guidance is yet to be determined. Further, the specific role of TAZ in mediating contact guidance is currently very speculative and has therefore not been included in the proposed model. However, HSP90 appears to play a role in differentially governing the response to biomimetic size scale features that is abrogated by YAP and TAZ. Additionally, this study further supports the potency of biophysical cues in cellular regulation and the importance of cell and tissue specific investigations of core signaling pathways.

## Supporting Information

Figure S1 **Relative changes in protein expression on planar and patterned surfaces.** Representative Western blots and their corresponding optical densities (O.D.) normalized to β-actin are shown. O.D. corresponding to planar samples was designated as 1 and those on patterned surfaces were expressed relative to planar samples (Lane 1– Planar, Lane 2–400 nm, Lane 3–1400 nm and Lane 4–4000 nm). When performed in triplicate, no statistically significant differences were observed in protein expression between planar and patterned surfaces. Although, YAP-H9 trended to be expressed lower on 400 nm surfaces while pYAP trended to be expressed higher on 4000 nm surfaces. TAZ remained unaltered on all surfaces.
(TIF)

Figure S2 **Simultaneous knockdown of YAP, TAZ and treatment with 17-AAG significantly inhibited cell viability in corneal epithelial cells.** Knockdown of YAP and/or TAZ trended to reduce cell viability. This inhibition of cell viability was exaggerated with 17-AAG treatment, especially after simultaneous knockdown of YAP and TAZ. Statistical comparisons were performed using Kruskal-Wallis pairwise multiple comparison, ***p<0.001 compared with Control cells and ###p<0.001 compared with siCtrl cells.
(TIF)
