# Supplementary figures and images for: Involvement of YAP, TAZ and HSP90 in Contact Guidance and Intercellular Junction Formation in Corneal Epithelial Cells
Source: PLoS One. 2014 Oct 7;9(10):e109811. doi: 10.1371/journal.pone.0109811 (PMC4188597; doi:10.1371/journal.pone.0109811)

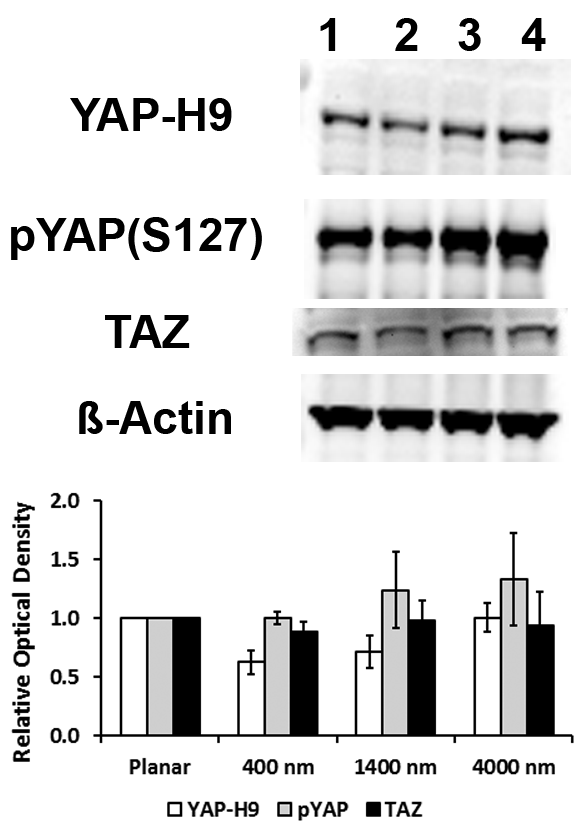

Supplement: Figure S1 — Relative changes in protein expression on planar and patterned surfaces. Representative Western blots and their corresponding optical densities (O.D.) normalized to β-actin are shown. O.D. corresponding to planar samples was designated as 1 and those on patterned surfaces were expressed relative to planar samples (Lane 1– Planar, Lane 2–400 nm, Lane 3–1400 nm and Lane 4–4000 nm). When performed in triplicate, no statistically significant differences were observed in protein expression between planar and patterned surfaces. Although, YAP-H9 trended to be expressed lower on 400 nm surfaces while pYAP trended to be expressed higher on 4000 nm surfaces. TAZ remained unaltered on all surfaces. (TIF) [file pone.0109811.s001.tif]

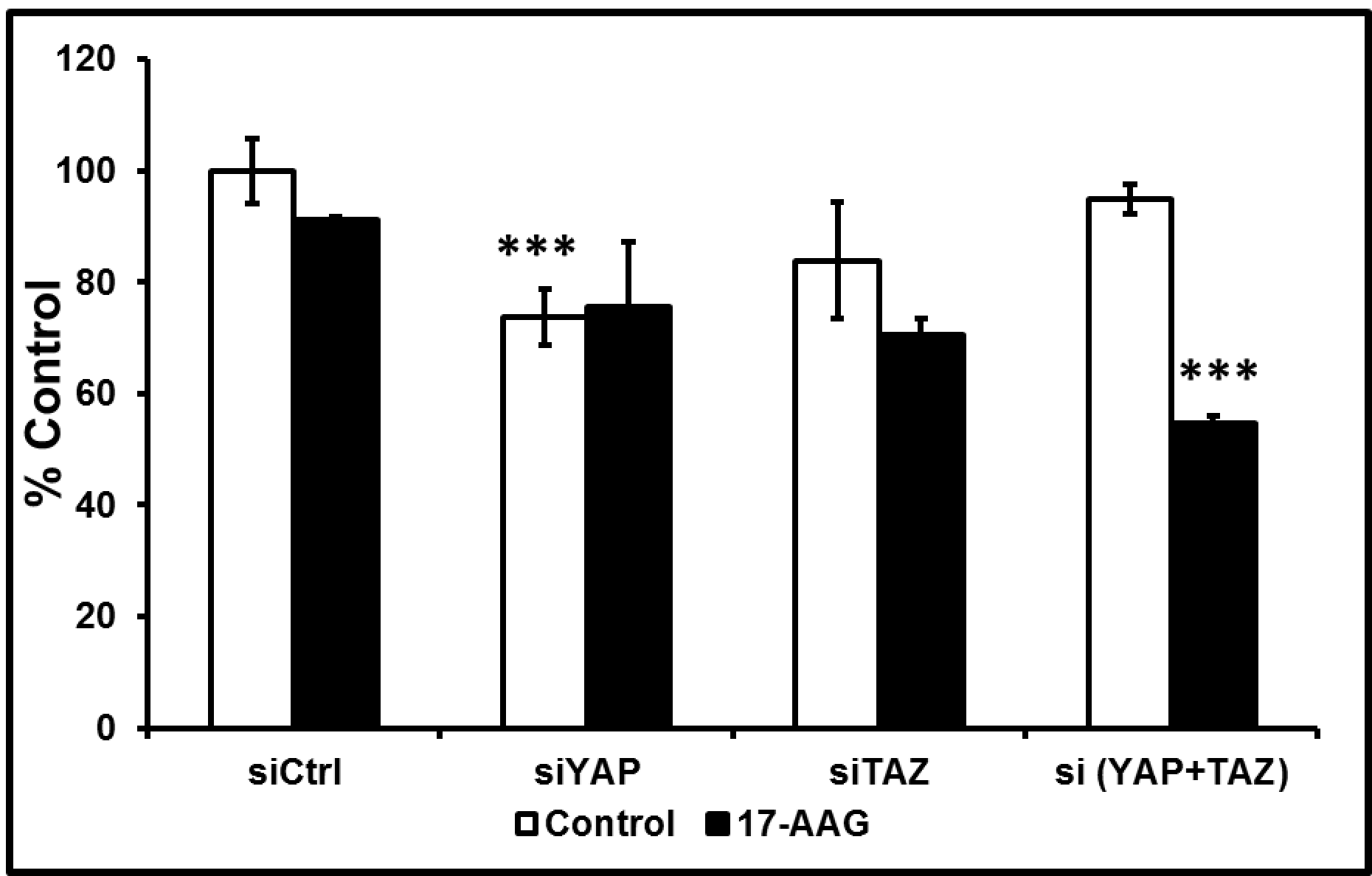

Supplement: Figure S2 — Simultaneous knockdown of YAP, TAZ and treatment with 17-AAG significantly inhibited cell viability in corneal epithelial cells. Knockdown of YAP and/or TAZ trended to reduce cell viability. This inhibition of cell viability was exaggerated with 17-AAG treatment, especially after simultaneous knockdown of YAP and TAZ. Statistical comparisons were performed using Kruskal-Wallis pairwise multiple comparison, ***p<0.001 compared with Control cells and ###p<0.001 compared with siCtrl cells. (TIF) [file pone.0109811.s002.tif]
